# Pyroptosis in Alopecia Areata: Synthesizing Emerging Hypotheses and Charting a Path to New Therapies

**DOI:** 10.3390/biomedicines13122940

**Published:** 2025-11-29

**Authors:** Mateusz Łysek, Justyna Putek, Beata Jastrząb-Miśkiewicz, Jacek C. Szepietowski, Piotr K. Krajewski

**Affiliations:** 1 Department of Dermatology, Research and Development Center, Regional Specialist Hospital, 51-124 Wroclaw, Poland; lysek.mateusz@gmail.com (M.Ł.); putek.justyna@gmail.com (J.P.); 2 Division of Dermatology, Venereology and Clinical Immunology, Faculty of Medicine, Wroclaw University of Science and Technology, Grunwaldzki Sq. 11, 51-377 Wroclaw, Polandjacek.szepietowski@pwr.edu.pl (J.C.S.); 3 Department of Dermato-Venereology, 4th Military Hospital, 50-981 Wroclaw, Poland; 4 Lübeck Institute of Experimental Dermatology, University of Lübeck, 23562 Lübeck, Germany

**Keywords:** alopecia areata, inflammatory dermatoses, pyroptosis, inflammasome, NLRP3, inflammation

## Abstract

**Background/Objectives**: Alopecia areata (AA) is a common, noncicatricial autoimmune hair loss disorder characterized by relapsing inflammation and breakdown of hair follicle immune privilege. Increasing amounts of evidence suggest that pyroptosis, a lytic and inflammatory form of programmed cell death mediated by gasdermins and inflammasome activation, may play a role in AA pathogenesis. This review aims to synthesize current data on the molecular mechanisms linking inflammasome-driven pyroptosis with AA and to highlight emerging therapeutic opportunities. **Methods**: A comprehensive literature review was conducted focusing on mechanistic studies, ex vivo human scalp models, murine AA models, and interventional clinical data. A structured system of Levels of Evidence (LoE) and standardized nomenclature for experimental models was applied to ensure transparency in evaluating the role of pyroptosis and treatment strategies in AA. **Results**: Available evidence indicates that outer root sheath keratinocytes express functional inflammasome components, including NOD-like receptor family, pyrin domain containing 3 (NLRP3), adaptor-apoptosis-associated-speck-like protein (ASC), and caspase-1, and contribute to interleukin (IL)-1β release and pyroptotic cell death. Mitochondrial dysfunction, mediated by regulators such as *PTEN* and *PINK1*, amplifies NLRP3 activation and cytokine secretion, linking mitophagy impairment with follicular damage. Animal and human biopsy studies confirm increased inflammasome activity in AA lesions. Therapeutic approaches targeting pyroptosis include Janus kinase (JAK) inhibitors, biologics, Phosphodiesterase 4 (PDE4) inhibitors, mesenchymal stem cell therapy, natural compounds, and inflammasome inhibitors such as MCC950. While some agents demonstrated efficacy in clinical trials, most strategies remain at preclinical or early clinical stages. **Conclusions**: Pyroptosis represents a critical mechanism driving hair follicle structural and functional disruption and immune dysregulation in AA. By integrating evidence from molecular studies, disease models, and early clinical data, this review underscores the potential of targeting inflammasome-driven pyroptosis as a novel therapeutic strategy.

## 1. Introduction

Alopecia areata (AA) is a noncicatricial autoimmune hair loss disorder. The degradation of the hair follicle’s immune privilege results in chronic lymphocytic inflammation [1]. It can be clinically distinguished by sharply demarcated patches of alopecia on the scalp; however, sometimes it can progress to extensive or even complete hair loss. The prevalence of AA is around 2% [2,3,4]. The illness is characterized by relapsing course and is associated with an increased risk of other comorbidities such as vitiligo, autoimmune hypothyroidism, systemic lupus erythematosus, and psoriasis [5,6,7]. Although the pathogenesis of AA is not fully understood, psychological stress is considered as a potential environmental trigger. However, stress alone cannot explain all the cases, as AA sometimes affects newborns and infants [8,9]. Some additional factors such as infections or toxic exposures can also be involved in the autoimmune dysregulation and be involved in the pathogenesis of AA [10].

Pyroptosis is an inflammatory form of programmed cell death mediated by proteins called gasdermins, which are involved in pore formation, and are stimulated by inflammasome activation [11]. Unlike apoptosis, which is not associated with immunological events, in pyroptosis, proinflammatory cytokines, such as interleukin-1β (IL-1β) and IL-18 are released [11]. Increasing evidence suggests that pyroptosis may play a role in the pathogenesis of inflammatory and autoimmune diseases, including AA, becoming an interesting therapeutic target [12,13].

Despite numerous studies on the topic, the pathophysiology of AA remains only partially understood. Current therapies are often nonspecific, with variable efficacy and risk of relapse. Pyroptosis has emerged as a potential mechanism, which underlies the follicular functional disruption and provokes the inflammation in AA [13]. However, this aspect of the disease has received little attention in the literature and, at present, it remains mainly a hypothesis-generating concept. To date, no comprehensive review has analyzed the available evidence on the role of pyroptosis in AA. This review aims to fill this gap by examining the molecular links between inflammasome activation, pyroptotic cell death, and the immunopathogenesis of AA, and tries to open new possibilities in the therapy for treating AA. Nevertheless, it should be noted that this review serves more as a theoretical model, which provides a conceptual framework for future studies.

## 2. Pyroptosis—A Lytic and Inflammatory Type of Programmed Cell Death

The balance between cellular proliferation and death is fundamental for maintaining tissue homeostasis in multicellular organisms [12]. There are two types of cell death: non-programmed or programmed cell death (PCD) [12]. Pyroptosis is a form of programmed cell death which initially was regarded as a subtype of apoptosis. However, subsequent studies demonstrated that pyroptosis is mechanistically different [14,15]. While apoptosis is characterized by nuclear condensation, membrane integrity, and controlled breakdown of cellular components, pyroptosis involves pore formation in the plasma membrane, cellular swelling, rupture, and the release of cytoplasmic content [16,17]. This uncontrolled discharge of molecules, particularly IL-1β and IL-18, enhances inflammation and activates innate and adaptive immune pathways [18,19].

The discovery of gasdermin D (GSDMD) was a milestone in understanding the molecular basis of pyroptosis [20]. Cleavage of GSDMD by caspase-1 releases its N-terminal fragment, which inserts into membranes and generates pores, which drives cell lysis [20,21]. The human gasdermin family includes six members (GSDMA, GSDMB, GSDMC, GSDMD, GSDME/DFNA5, and PJVK/DFNB59) [12]. After activation by microbial signals or immune proteases such as caspases and granzymes, the inhibitory interaction is relieved, enabling the N-terminal fragment to perforate membranes [22,23]. Pyroptosis is closely associated with the innate immune system. Pattern recognition receptors (PRRs) detect microbial products (PAMPs), host-derived danger signals (DAMPs), or stress-induced alterations, initiating signaling cascades that upregulate cytokines and mobilize protective responses, such as autophagy, to eliminate damaged organelles [24]. One of the best-studied signaling complexes is the inflammasome, a multiprotein structure typically composed of a sensor (i.e., NOD-like receptor family, pyrin domain-containing 3 (NLRP3)), the adaptor apoptosis-associated speck-like protein containing a caspase-recruiting domain (ASC), and the effector protease caspase-1 [11,12]. Upon activation by infection or cellular stress, caspase-1 undergoes autoproteolysis, leading to the maturation of IL-1β and IL-18, as well as the cleavage of GSDMD, which results in pore formation and finally pyroptosis [11].

Physiologically, pyroptosis serves as a host defense mechanism by eliminating infected cells and alarming the immune system. However, when dysregulated or excessive, it causes uncontrolled inflammation, extensive cell loss, and tissue damage, contributing to the pathogenesis of inflammatory and autoimmune disorders.

## 3. Signaling Pathways in Pyroptosis

Currently, four distinct signaling pathways are described to trigger pyroptosis: the canonical inflammasome pathway, the non-canonical inflammasome pathway, pathways driven by granzymes and caspase-dependent apoptotic signaling [12]. The second and the last pathway appears to have minimal relevance to the pathogenesis of AA and will therefore not be discussed in this review. Regardless of the upstream trigger, the execution phase is carried out by gasdermin proteins, which must undergo proteolytic cleavage by caspases or granzymes to form membrane pores [12]. Caspases can be broadly grouped into two functional categories: inflammatory and apoptotic [25]. The inflammatory subgroup (caspase-1, -4, -5, and -11) is central to the innate immune response, since it not only initiates pyroptotic cell death to limit intracellular pathogen survival but also processes precursor cytokines into their active forms [26]. Within the canonical pathway, caspase-1 is recruited and activated by inflammasome complexes. In contrast, caspases-4, -5, and -11 omit such complexes, because they are directly activated through binding to bacterial lipopolysaccharide (LPS) [12]. Apoptotic caspases are known for coordinating the controlled degradation of cells during apoptosis [27]. However, they also have been found to cleave gasdermins, which links apoptosis machinery to pyroptotic responses [27].

### 3.1. Canonical and Non-Canonical Inflammasome Pathway

The first described signaling pathway of pyroptosis was the canonical inflammasome pathway. In this mechanism, microbial components, PAMPs, are recognized by PRRs, such as toll-like receptors and members of the NOD-like receptor family (NLRP, including NLRP1, NLRP3, and NLRC4) [24,28,29]. Besides PAMPs, cells under stress or damage release endogenous molecules called DAMPs, which are also detected by PRRs. These receptors are integral components of the inflammasome complex [24]. Their role is to sense danger signals and initiate inflammation. Well-characterized inflammasome sensors responds to different triggers such as bacteria, viruses, fungi, uric acid crystals, reactive oxygen species, ATP, or double-stranded DNA of microbial or viral origin [30]. Once a receptor is engaged, pro-caspase-1 is recruited and activated through a process of autoproteolysis. Caspase-1 then converts pro-IL-1β and pro-IL-18 into their mature forms and cleaves gasdermin D, whose N-terminal fragment forms membrane pores in the cell [31].

**Figure 1:**
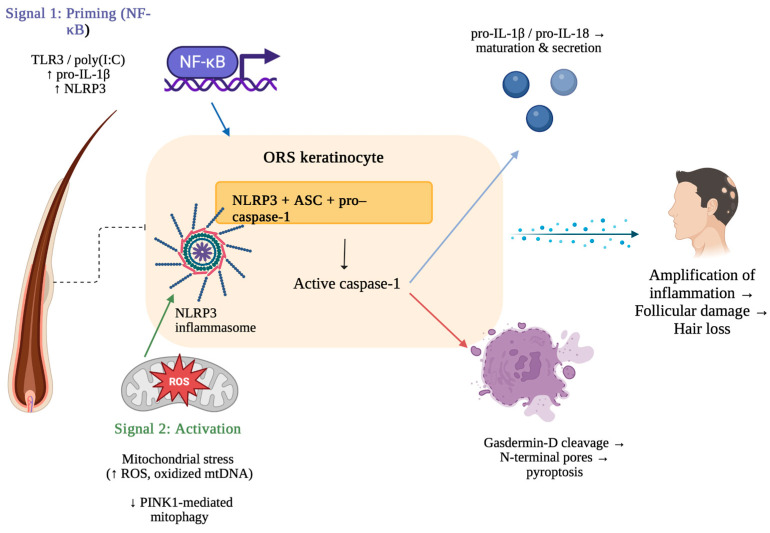
Mechanism of NLRP3 inflammasome activation and pyroptosis in alopecia areata. Created in BioRender. Krajewski, P. (2025) https://BioRender.com/3cl1t7i (accessed on 10 August 2025).

The Nuclear Factor kappa-light-chain enhancer of activated B cells (NF-κB) pathway represents a central regulatory mechanism in pyroptosis, because it controls the transcriptional priming of inflammasome components [32]. Following the recognition of PAMPs or DAMPs by Toll-like receptors (TLRs), intracellular signaling cascades are initiated through adapter proteins [6,33]. This leads to the activation and nuclear translocation of NF-κB, which drives the expression of key inflammasome components, including *NLRP3*, pro-IL-1β, and pro-IL-18 (Figure 1). This “priming step” (signal 1) prepares the cell for inflammasome structure by elevating protein levels and inducing post-translational modifications of *NLRP3* (Figure 1) [34,35,36,37]. The following “activation step” (signal 2), triggered by additional PAMPs or DAMPs, then promotes the structure of the inflammasome complex, caspase-1 activation, and finally leads to pyroptotic cell death (Figure 1) [32].

The process begins with Signal 1 (priming), in which NF-κB activation induced by TLR3 or poly (I:C) upregulates pro-IL-1β and NLRP3 expression in outer root sheath (ORS) keratinocytes. Signal 2 (activation) is triggered by mitochondrial stress, characterized by increased ROS and oxidized mtDNA together with impaired *PINK1*-mediated mitophagy, resulting in the formation of the NLRP3 inflammasome. The inflammasome complex (NLRP3, ASC, pro–caspase-1) activates caspase-1, which promotes cleavage and secretion of IL-1β/IL-18 and cleavage of gasdermin D. Gasdermin D forms membrane pores, resulting in pyroptosis [38,39].

The non-canonical pathway acts independently of classical inflammasome formation. It is activated mostly by intracellular LPS from Gram-negative bacteria. This pathway has minimal relevance to pyroptosis in AA and therefore will not be discussed in this review.

### 3.2. Pyroptosis Triggered by Granzymes

Natural killer (NK) cells and cytotoxic T cells release granzymes that enter target cells through pores formed by perforin [40,41,42]. Once inside, these proteases can cleave members of the gasdermin family and initiate pyroptosis, particularly in cancer cells. Granzyme A (GZMA), the most frequently occurring serine protease in this group, has long been associated with programmed cell death. However, now this protease is also known to regulate inflammatory responses by promoting the release of cytokines, thereby favoring pyroptosis [43]. For example, GZMA secreted by cytotoxic T cells has been shown to cleave GSDMB, enabling pore formation in the plasma membrane and triggering pyroptotic death in GSDMB-positive tumor cells [44]. Similarly, granzyme B (GZMB) from NK cells targets GSDME, cleaving it at the same site recognized by caspase-3 [45]. This cleavage release the N-terminal effector fragment of GSDME, which inserts into the membrane and drives cell lysis [40].

## 4. The Role of Pyroptosis in Alopecia Areata

In AA, loss of hair follicle immune privilege triggers a chronic, lymphocytic inflammation resulting in autoimmune disorder [1]. At the molecular level, hair follicles (HFs) are normally protected as immune-privileged sites. This state is maintained by the low expression of major histocompatibility complex (MHC) class I molecules in the anagen bulb, which reduces recognition by CD8+ T cells. In addition, the local release of immunosuppressive mediators, i.e., transforming growth factor-β1 (TGF-β1), or IL-10 creates a specific environment, which can protect this privilege [4,46]. When this protection breaks down, hair follicles instead show upregulation of MHC class I and II molecules, induction of Natural Killer Group 2 Member D (NKG2D) ligands, and a decline in regulatory mediators such as TGF-β1, and IL-10 which all contribute to autoimmune inflammation [4,46,47].

In AA, there is widespread cytokine dysregulation involving Th1 (IL-2, IFN-γ, IL-12, TNF), Th2 (IL-4, IL-5, IL-6, IL-17E, IL-31, IL-33), and Th17 (IL-17, IL-17F, IL-21, IL-22, IL-23) pathways. Elevated levels of IL-2, IL-12, IL-17, IL-17E, and TNF correlate with disease severity and duration, supporting the concept that inflammation associated with inflammasomes can be a central mechanism of follicular structural and functional disruption [48]. Additionally, excessive production of proinflammatory mediators, including IFN-I and IFN-II, IL-15, and interferon-inducible chemokines (CXCL9, CXCL10, CXCL11), plays a key role by recruiting CXCR3+ effector T cells to the peribulbar region, where autoreactive lymphocytes attack follicular epithelial cells [4,47]. CD8+ NKG2D+ T cells further intensify this process by producing IFN-γ via the JAK1-STAT1 and JAK2-STAT2 pathways. IFN-γ, in turn, stimulates IL-15 secretion in follicular epithelial cells. Then, IL-15 signals back to CD8+ NKG2D+ T cells through JAK1 and JAK3, strengthening IFN-γ production and creating a positive feedback loop [4,49,50].

It is worth underlining that IL-15 is considered to be one of the most important interleukins in the pathogenesis of AA [51,52]. Higher serum levels of IL-15 have been found in AA patients compared to healthy controls and are correlated with a more severe course of the disease and worse prognosis [52]. Additionally, IL-15 activates key immune cells involved in the pathogenesis of AA, and its recombinant form can promote human scalp hair follicle growth and reduce apoptosis in hair matrix keratinocytes ex vivo [53]. Moreover, it was found that supplementation with the recombinant form of IL-15 can prevent IFN-γ-induced collapse of hair follicle immune privilege, supporting the potential therapeutic role of IL-15 [53].

Previous studies demonstrated that IL-1β is notably elevated in AA lesions, which may disrupt the normal hair cycle and thereby contribute to hair follicle degeneration [4]. Of note, keratinocytes, which are not professional immune cells, represent a significant source of IL-1 in the skin [46,54,55]. These cells, particularly those within the outer root sheath (ORS) of hair follicles, appear to function as immunocompetent cells that actively participate in the autoinflammatory processes characteristic of AA [56].

The study by Shin et al. [56] provided the first direct evidence that ORS cells constantly express inflammasome components, including NLRP3, ASC, and caspase-1, and were capable of forming fully functional inflammasomes. In scalp biopsies from AA patients, immunohistochemistry revealed strong expression of NLRP3, ASC, and caspase-1 in the ORS of hair follicles as well as in infiltrating immune cells [56]. Yet, these proteins were only weakly expressed in normal scalp skin [56]. Similarly, in a C3H/HeJ mouse model of AA, inflammasome proteins were highly upregulated in ORS cells and inflammatory infiltrates of affected skin [57]. These findings demonstrated that ORS cells themselves, rather than immune infiltrates alone, can act as sources of cytokines associated with pyroptosis. This expands the concept of AA from being purely a T cell-driven autoimmune disease to also autoinflammatory mechanisms driven by the innate immune system [57].

Furthermore, stimulation of ORS cells with poly(I:C)—a synthetic RNA analog and TLR3 ligand—significantly increased mRNA and protein levels of inflammasome components (NLRP3, ASC, CASP1, IL1B) [56]. Additionally, it increased secretion of active caspase-1 and IL-1β and promoted co-localization of NLRP3 with ASC [56]. Importantly, silencing caspase-1 or NLRP3 using microRNA notably reduced poly(I:C)-induced IL-1β release, confirming that cytokine secretion depended on inflammasome activation [56]. Of note, the NF-κB pathway was identified as a key mediator of poly(I:C)-induced IL-1β production in cultured ORS cells [32].

Moreover, recent work has revealed that mitochondrial quality control mechanisms are also linked to inflammasome activity in AA. Specifically, *PTEN* (phosphatase and tensin homolog) and *PINK1* (*PTEN*-induced kinase 1), a key regulator of mitophagy, have been shown to influence pyroptosis through their effects on mitochondrial function [58]. In AA scalp tissue and IFN-γ/poly(I:C)-stimulated ORS cells, mitochondrial DNA damage and elevated mitochondrial reactive oxygen species (ROS) were observed [58]. Both of them promote NLRP3 activation [58]. Activating PINK1-driven mitophagy reduced inflammasome activity, while silencing PINK1 increased NLRP3 signaling and cytokine release [58]. These findings highlight *PTEN*/*PINK1*-driven mitophagy as a key negative regulator of pyroptosis in ORS cells and show that mitochondrial dysfunction can be a potential therapeutic target in AA [58]. The comparison of classical and pyroptosis-driven mechanism in AA is given in Table 1 and key cytokines and pathways are summarized in Table 2.

Although these findings provide valuable insights into the potential role of pyroptosis in AA, the current evidence remains limited and, largely hypothetical. Not all proposed mechanisms are proven by strong experimental and clinical data, and still the precise mechanism of pyroptosis is not fully understood. Therefore, these concepts should be viewed as early, hypothesis-generating ideas rather than established pathomechanisms. Clarifying these facts can be crucial in developing new therapies.

## 5. New Therapies of AA Based on Pyroptosis and Future Directions

Pyroptosis seems to be one of the mechanisms inducing hair follicle damage and maintaining the autoimmune response in AA, which opens up new perspectives for treatment strategies. Current research is focused on targeting pyroptosis and related inflammatory pathways, with the aim of reducing cytokine release and limiting inflammation.

To ensure a transparent evaluation of the strength of available data, we applied a custom, structured nomenclature of Levels of Evidence (LoE). The following categories were used:

- LoE-H2: Interventional clinical data in humans, including randomized and non-randomized clinical trials. The study phase (e.g., phase II or III) is specified where applicable.
- LoE-H1: Observational human data, such as analyses of patient biopsies, single-cell RNA sequencing (scRNA-seq), proteomics/serology, or immunohistochemistry (IHC).
- LoE-A2: In vivo animal studies using AA-specific models (e.g., C3H/HeJ mice with spontaneous or graft-induced AA, humanized or xenograft AA models).
- LoE-A1: In vivo animal studies using non-AA-specific hair growth or hair cycle models (e.g., C57BL/6 telogen-to-anagen induction), which assess follicular biology without autoimmune context.
- LoE-X: Ex vivo human models, including organotypic skin cultures, isolated hair follicles, or other patient-derived tissue explants.
- LoE-C: In vitro cellular studies, including cultured human outer root sheath (ORS) keratinocytes, keratinocyte lines, or co-culture systems.

The standardized nomenclature for experimental models was also applied. The following labels were used throughout the manuscript:

- Model-AA-C3H, murine alopecia areata model (C3H/HeJ, spontaneous or graft-induced AA)
- Model-HG-C57, murine hair growth model (C57BL/6, telogen-to-anagen induction, non-autoimmune)
- Model-ORS-polyIC, cultured human outer root sheath keratinocytes stimulated with poly(I:C) ± IFN-γ/TNF
- Model-Skin-ExVivo, human scalp skin organotypic cultures or biopsies maintained ex vivo
- Model-Human-Biopsy, in vivo human scalp biopsy material
- Model-Trial-AA, interventional clinical trial in patients with alopecia areata.

**Figure 2:**
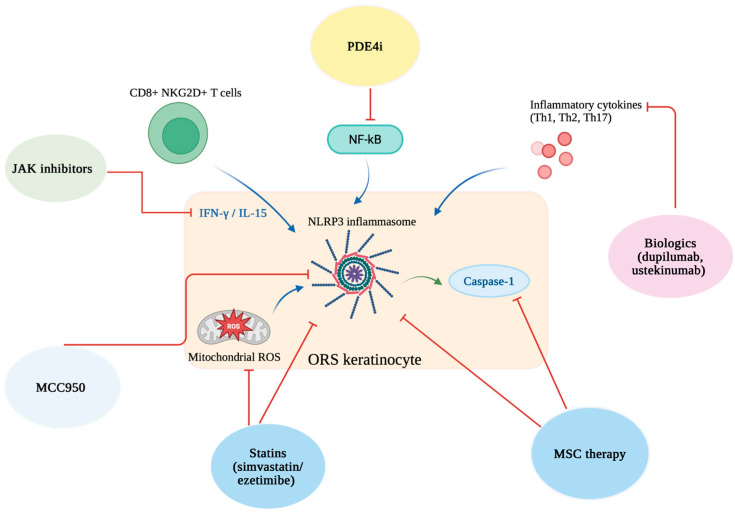
Schematic representation of therapeutic strategies targeting different stages of pyroptosis in alopecia areata. Created in BioRender. Krajewski, P. (2025) https://BioRender.com/gh3jl05 (accessed on 10 August 2025).

By using these two systems, we wanted to ensure that the review would be transparent, precise and reliable based on the available literature. The schematic effects of drugs on the different stages of pyroptosis are illustrated in Figure 2.

JAK inhibitors reduce IFN-γ/IL-15 signaling from CD8^+^ NKG2D^+^ T cells; PDE4 inhibitors block NF-κB activation; biologics (e.g., dupilumab, ustekinumab) suppress inflammatory cytokines; MCC950 inhibits the NLRP3 inflammasome; statins (simvastatin/ezetimibe) reduce mitochondrial ROS; mesenchymal stem cell (MSC) therapy limits NLRP3 inflammasome activation and caspase-1 activity in ORS keratinocytes.

### 5.1. AA-Specific Interventions

#### 5.1.1. JAK Inhibitors (LoE-H2; Model-Trial-AA/LoE-A2; Model-AA-C3H)

Advances in the molecular understanding of AA have highlighted the importance of the JAK-STAT signaling pathway in disease progression [4,59]. The JAK family includes *JAK1*, *JAK2*, *JAK3*, and *TYK2*, which transmit signals from cytokine receptors and lead to the phosphorylation of STAT. Then, STAT moves into the nucleus and activates the transcription of proinflammatory genes [60,61]. Cytokines such as IFN-γ and IL-15, which are crucial to AA pathogenesis, depend on this pathway, which makes JAK inhibitors (JAKi) reliable therapeutic agents (Figure 2) [60,61]. Preclinical studies using the C3H/HeJ mouse model demonstrated that oral JAKi, including ruxolitinib and tofacitinib, could prevent and reverse AA, supporting their clinical potential [62,63,64].

Importantly, since IFN-γ-driven JAK-STAT activation prepares inflammasome components and induces IL-1β production, JAK inhibition may indirectly block pyroptotic pathways in hair follicle cells [65]. By reducing upstream cytokine signaling, JAKi lower the inflammatory environment required for inflammasome building and caspase-1 activation, which can potentially limit follicular damage induced by pyroptosis [65]. Although topical JAKi seem to reduce systemic adverse effects, their efficacy remains variable, and further studies are needed to clarify their role not only in immune modulation but also in the control of inflammasome-driven pyroptosis in AA [66,67].

The anti-inflammatory action of JAK inhibitors is relatively non-specific, as the targeted signaling pathways are shared by numerous cytokines [4,68]. Baricitinib is a JAK1/JAK2 inhibitor, and its efficacy and safety were confirmed in clinical trials: BRAVE-AA1 and BRAVE-AA2 [69,70]. Similarly, deuruxolitinib, which is also a JAK1/JAK2 inhibitor, achieved significant improvements in hair regrowth, as demonstrated in the THRIVE-AA1 trial [71]. The newest generation of JAK inhibitors, such as brepocitinib (JAK1/TYK2 inhibitor) and ritlecitinib (JAK3/TEC inhibitor), have improved selectivity and are under clinical investigation in AA [72,73]. Currently, the only two medications approved by the European Medicines Agency for alopecia areata are baricitinib for adults and ritlecitinib for individuals aged 12 years and older. In addition, in the United States, deuruxolitinib was approved by the U.S. Food and Drug Administration in July 2024 for adults.

Evidence for JAK inhibitors in AA ranges from AA-specific murine models to phase II and III clinical trials. This evidence positions JAKi as the most advanced pyroptosis-modulating strategy to date, and one of the most effective treatments for AA.

#### 5.1.2. Biologics (LoE-H2/H1; Model-Trial-AA/Model-Human-Biopsy)

Biologic agents targeting the Th1, Th2, and Th17 axes may influence pathways leading to pyroptosis in AA (Figure 2) [4,74,75]. Cytokines such as IFN-γ, IL-12, and IL-23 are known to strengthen JAK-STAT signaling and promote inflammasome activation. By blocking these cytokines, indirectly the level of IL-1β and IL-18 can be reduced and so can the pyroptotic cell death within hair follicles [74,75].

Dupilumab, a monoclonal antibody that targets IL-4Rα and thereby blocks IL-4/IL-13 signaling, has demonstrated therapeutic activity in AA [76]. In a clinical trial, dupilumab stimulated hair regrowth, with higher prediction in patients with elevated IgE [76]. On the other side, dupilumab was also described to induce AA in some case reports [23]. Additionally, numerous case reports suggest that ustekinumab, an antibody targeting IL-12/23, can also promote hair regrowth in AA [77,78]. However, these observations are rather limited to case reports and often involve patients with comorbidities such as psoriasis [79].

Hypothetically, as IL-1β remains a central cytokine generated during inflammasome activation, the potential use of IL-1 inhibitors such as anakinra or canakinumab should be evaluated in the future. For example, anakinra demonstrated a significant reduction in inflammatory lesions and pain scores in patients with hidradenitis suppurativa, a condition whose pathogenesis is also partially driven by pyroptosis [79].

Currently, the evidence for biologics in AA is heterogeneous, ranging from controlled trials to case series. Biopsy studies suggest that targeting Th1/Th2/Th17 cytokines could be useful, but clinical results are inconsistent, showing the need for larger trials to define their real role in the AA.

#### 5.1.3. Phosphodiesterase 4 Inhibitors (LoE-H2; Model-Trial-AA)

Apremilast and crisaborole belong to a group of phosphodiesterase 4 (PDE4) inhibitors. They modulate the innate immune system by increasing intracellular cyclic adenosine monophosphate (cAMP) levels, which in turn suppress NF-κB signaling [4,80]. NF-κB is involved in pyroptosis, so it suggests that PDE4 can limit inflammasome-driven inflammation in hair follicle cells (Figure 2) [80]. However, the clinical efficacy of PDE4 inhibitors in AA remains variable [81,82,83] and is limited to pilot clinical trials and case reports, with mixed outcomes.

#### 5.1.4. Other AA-Linked Modulators (LoE-H2/H1)

Some therapeutic agents used in AA may influence pyroptosis indirectly by modulating upstream inflammatory pathways. For example, abatacept, which was evaluated in an open-label clinical study, blocks T-cell costimulation and reduces the release of IFN-γ and TNF-α [84]. That in the end, limits signals which drive inflammasome activation [84]. Further, platelet-rich plasma (PRP) has been assessed in a randomized controlled trial in patchy AA [85,86]. Anti-inflammatory effect of PRP was assigned to suppression of MCP-1 and induction of TGF-β, which are mechanisms that may reduce the inflammation promoting pyroptosis [85,86]. Statins, particularly simvastatin, have been investigated in small clinical series and appear to inhibit NF-κB and JAK/STAT signaling (Figure 2) [87,88,89]. Additionally, they reduce ROS generation. All of these are key triggers of *NLRP3* activation [87,88,89]. Similarly, antihistamines such as fexofenadine and ebastine, supported mainly by retrospective clinical data, decrease Th2 cytokines, IFN-γ, and substance P, thereby lowering T-cell infiltration around hair follicles [90]. The above mechanisms suggest possible indirect effects, however direct evidence linking these agents to pyroptosis in AA remains limited.

Since 1979, dithranol (anthralin) has been used as a topical therapy for AA [91]. With the recent advances in understanding pyroptosis, its mechanism of action can now be viewed from a new perspective. Topical dithranol induces localized inflammation and disrupts the skin barrier, leading to epidermal hyperproliferation [92]. This irritation promotes the release of antimicrobial peptides (S100a8/a9) and proinflammatory cytokines such as IL-1β, both closely associated with pyroptosis pathways [92]. Understanding how dithranol modulates IL-1β secretion and inflammasome activity may open new perspectives for treatment strategies that specifically target pyroptosis in AA.

#### 5.1.5. Mesenchymal Stem Cell Therapy (LoE-X; Model-Skin-ExVivo/LoE-A2; Model-AA-C3H)

Human outer root sheath cells (hORSCs), which are integral to hair follicle structure and immune regulation, were shown to constantly express NLRP3 inflammasome components [93]. When stimulated with IFN-γ, hORSCs exhibited marked upregulation of NLRP3 and related inflammasome markers, showing a higher tendency for pyroptosis. Importantly, treatment with mesenchymal stem cell therapy (MSCT) effectively suppressed this response, reducing inflammasome activation and caspase-1–mediated pyroptotic signaling [93]. Notably, these effects were comparable to, or stronger than, those observed with ruxolitinib, highlighting the potential of hMSCs to limit follicular damage [94].

Although promising results have been observed in vitro, their effectiveness in vivo remains limited. Another study evaluated the efficacy of locally administered MSCT in an AA mouse model induced by IFN-γ [95]. Compared to controls, mice treated with MSC exhibited enhanced hair regrowth. MSCT significantly reduced local inflammatory cytokines, including JAK1, JAK2, STAT1, STAT3, IFN-γR, IL-1β, IL-16, IL-17α, and IL-18, while systemic cytokine levels remained unaffected (Figure 2) [95]. Additionally, MSC treatment normalized the expression of Wnt/β-catenin pathway genes and fibroblasts growth factors (*FGF7*, *FGF2*), which are essential for hair cycle regulation [95]. The available evidence on MSCT in AA is based on a combination of ex vivo human outer root sheath cell experiments and disease-relevant murine models (C3H/HeJ AA). Together, these levels of evidence suggest promising but still preliminary therapeutic potential, and further controlled human trials are needed to prove their efficacy.

#### 5.1.6. Natural Compounds in AA (LoE-A2; Model-AA-C3H)

Currently, there are many different substances and compounds whose therapeutic effects are being studied in AA. In a mouse model (C3H/HeJ strain), researchers showed that pharmacological inhibition of the *NLRP3/caspase-1/GSDMD* pathway with MCC950 led to a marked improvement of disease manifestations (Figure 2) [96]. Oral administration of the MCC950 inhibitor not only suppressed inflammasome components (*NLRP3*, *ASC*, *caspase-1 p10*, *GSDMD*) and proinflammatory cytokines (TNF-α, IL-6, IL-1β, IL-18), but also resulted in visible healing of skin lesions and regrowth of hair [96]. The severity of follicular structural and functional disruption was significantly reduced, and histological analysis confirmed a lower infiltration of immune cells and a higher number of regenerated hair follicles in the group treated with MCC950 [96]. Further, a natural anti-inflammatory agent, called total glucosides of paeony (TGP), provided comparable results in a concentration-dependent manner, decreasing both caspase-1 activity and pyroptotic cell death in skin tissue in AA mice [13]. Subsequent compounds called water-soluble Ginkgo biloba leaf polysaccharides (WGBPs) were shown to reduce inflammation and pyroptosis in an AA mouse model [97]. The acidic fraction WGBP-A2 increased vascular endothelial growth factor (VEGF) and hepatocyte growth factor (HGF) in skin, while the purified polysaccharide WGBP-A2b suppressed NF-κB–mediated inflammatory markers, such as TNF-α or IL-1β [97]. Additionally, Xuefu Zhuyu decoction (XZD) has been shown to induce hair regeneration in both AA patients and C3H/HeJ mice, accompanied by a marked reduction in proinflammatory cytokines such as IL-6, IL-1β, and TNF-α [98]. Since IL-1β and IL-18 are key mediators of inflammasome-driven pyroptosis, the suppression of upstream inflammatory signals by XZD suggests it may inhibit pyroptotic cell death in hair follicles [98]. To sum up, evidence in this category derives from AA-specific murine models, which are considered the gold standard for preclinical mechanistic and therapeutic studies in AA. Despite the fact that findings at this level provide strong experimental support, they remain preclinical, requiring validation in human studies.

### 5.2. Non-AA-Specific/General Hair-Growth Interventions (LoE-A1; Model-HG-C57/LoE-C; Model-ORS-polyIC)

SCD-153, a lipophilic prodrug of 4-methyl itaconate (4-MI), was designed to enter the skin and cells more easily, which can be used in alopecia areata therapy [99]. After topical application, SCD-153 is converted to 4-MI within the skin, minimizing systemic exposure [99]. In vitro studies on human keratinocytes stimulated with poly I:C or IFN-γ demonstrated that SCD-153 significantly reduced the expression of IL-1β, IL-6, TLR3, and IFNβ. In C57BL/6 mice, topical SCD-153 promoted hair growth more effectively than 4-MI, dimethyl itaconate, vehicle, or even the JAK inhibitor tofacitinib, showing promising indication in AA [99]. However, the evidence for this compound remains restricted to single in vitro and non-AA-specific murine studies, with no clinical trials conducted to date.

The overview of all therapies targeting pyroptosis in AA can be found in Table 3.

## 6. Conclusions

Pyroptosis may play a role in the pathogenesis of AA, which provides new insights into the disease beyond classical T cell-driven models. Evidence suggests that outer root sheath cells act as active participants in inflammasome signaling and cytokine release, while mitochondrial dysfunction acts differently and can intensify pyroptosis. These discoveries not only deepen our understanding of AA’s pathophysiology but also open novel therapeutic strategies. Preclinical clinical data support the potential of inflammasome inhibitors, natural anti-inflammatory compounds, mesenchymal stem cell therapy, JAK inhibitors, biologics, and PDE4 modulators to reduce pyroptosis and restore immune tolerance within the hair follicle; however, most of these strategies remain experimental. Moreover, current data on the involvement of pyroptosis in AA remain hypothetical, being based mainly on preclinical studies, with no interventional clinical studies available. Eventually, targeting pyroptosis could be a new way to reduce disease relapse, protect hair follicles, and support lasting hair regrowth in AA patients in the future.

## Figures and Tables

**Table 1:** Comparison of the classical immune mechanism and pyroptosis-driven mechanism in alopecia areata.

Aspect	Classical AA Mechanism	Pyroptosis-Driven Mechanism
Main mechanism	Loss of the hair follicle’s immune privilege results in autoimmune attack by lymphocytes	Pyroptosis in follicular cells releases danger signals and cytokines that strengthen inflammation
Main triggers	Collapse of hair follicle immune privilege (loss of MHC I downregulation, decrease in local immunosuppressants: TGF-β, IL-10, α-MSH, IDO, VIP)	Cellular stress, mitochondrial dysfunction, PAMPs/DAMPs activating PRRs and inflammasomes (e.g., NLRP3)
Key cells	CD8^+^ NKG2D^+^ T cells; helper T cells (Th1/Th2/Th17)	ORS keratinocytes, infiltrating immune cells; innate sensors drive caspase-1 activity within follicles
Key signaling pathways	Positive feedback between IFN-γ and IL-15 which sustain T cell activation and follicular attack	Inflammasome activation (NLRP3/ASC/caspase-1) → pyroptotic pore formation (GSDMD) → cytokine release → amplification of inflammation
Effector molecules	Cytokines/chemokines (IFN-γ, IL-15, CXCL9/10/11) recruit and activate cytotoxic T cells around the hair bulb	Gasdermins (mainly GSDMD) form membrane pores after cleavage by caspase-1 → cell swelling and lysis
Most important cytokines	IFN-γ, IL-15, TNF-α, IL-2, IL-12, IL-17	IL-1β, IL-18
Type of cell death	Apoptosis/immune-mediated cytotoxicity (CD8+ T cell killing of follicular epithelium)	Programmed lytic death (pyroptosis): pore formation, swelling, rupture, release of intracellular contents
Hair follicle outcome	Premature end of anagen, peribulbar infiltrate, hair loss	Direct damage to ORS and surrounding tissue via pyroptotic pores and IL-1 family cytokines
Therapeutic implications	JAK inhibitors, biologics targeting Th1/Th2/Th17 cytokines, immunosuppressants	Inflammasome inhibitors (e.g., MCC950), antioxidants, mitophagy inducers (PINK1/PTEN), MSC therapy, novel small molecules

AA, alopecia areata; ASC, apoptosis-associated speck-like protein containing a CARD; CD, cluster of differentiation; CXCL, C-X-C motif chemokine ligand; DAMP, danger-associated molecular pattern; GSDMD, gasdermin D; IDO, indoleamine 2,3-dioxygenase; IFN-γ, interferon gamma; IL, interleukin; JAK, Janus kinase; MCC950, selective NLRP3 inflammasome inhibitor; MHC I, major histocompatibility complex class I; MSC, mesenchymal stem cell; α-MSH, alpha-melanocyte-stimulating hormone; NKG2D, natural killer group 2D; NLRP3, NOD-like receptor family pyrin domain containing 3; ORS, outer root sheath; PAMP, pathogen-associated molecular pattern; PINK1, PTEN-induced kinase 1; PRR, pattern recognition receptor; PTEN, phosphatase and tensin homolog; TGF-β, transforming growth factor beta; Th, T helper cell; TNF-α, tumor necrosis factor alpha; VIP, vasoactive intestinal peptide; Arrow (→): The arrow indicates the sequential cascade of events in the pyroptotic pathway.

**Table 2:** Summary of key cytokines and pathways involved in AA.

Cytokine/Pathway	Role in AA	Relation to Pyroptosis	Notes
IL-1β	Central proinflammatory cytokine; disrupts hair cycle, promotes follicular degeneration	Direct product of caspase-1 cleavage in NLRP3 inflammasome; major mediator of pyroptosis	Human biopsies and ORS studies showed upregulation
IL-18	Activates CD8^+^ and “virtual memory” T cells; strengthens IFN-γ production; promotes chronic inflammation	Produced by caspase-1 during pyroptosis	Elevated in serum and lesional skin of AA patients
Th1 axis (IL-2, IFN-γ, IL-12, TNF)	Correlates with severity; promotes cytotoxic T-cell activity around follicles	Strengthens priming for IL-1β/IL-18 production	–
Th2 axis (IL-4, IL-5, IL-6, IL-17E, IL-31, IL-33)	Part of mixed cytokine pattern in AA	Can indirectly affect inflammasome via NF-κB/JAK-STAT	Mixed clinical signals with Th2-targeting biologics
Th17/IL-23 axis (IL-17, IL-17F, IL-21, IL-22, IL-23)	Associated with disease activity	Supports the priming of inflammasome genes	–
IFN–γ/IL-15 → JAK-STAT	Forms a positive feedback loop between follicular epithelium and CD8^+^ NKG2D^+^ T cells; stimulates inflammation	IFN-γ activates inflammasome genes; JAK inhibitors reduce this effect	JAK inhibitors clinically effective in AA
TNF-α	Potent proinflammatory cytokine; contributes to follicular inflammation	Stimulates NF-κB signaling → activates NLRP3 inflammasome	Elevated in AA, but anti-TNF biologics showed limited efficacy
NF-κB pathway	Major regulator of inflammatory gene expression	Drives transcription of NLRP3, pro-IL-1β, pro-IL-18 (“signal 1” priming step)	–
NLRP3 inflammasome	Canonical inflammasome sensor → activates caspase-1	Central driver of pyroptosis in ORS cells and infiltrating immune cells	Upregulated in AA lesions and in mouse models
Mitophagy (PINK1/PTEN axis)	Mitochondrial quality control	Negative regulator of NLRP3 activation and pyroptosis	Loss of PINK1 increases inflammasome activity in ORS

AA, alopecia areata; CD, cluster of differentiation; IFN-γ, interferon gamma; IL, interleukin; JAK, Janus kinase; NF-κB, nuclear factor kappa B; NKG2D, natural killer group 2D; NLRP3, NOD-like receptor family pyrin domain containing 3; ORS, outer root sheath; PINK1, PTEN-induced kinase 1; PTEN, phosphatase and tensin homolog; STAT, signal transducer and activator of transcription; Th, T helper cell; TNF-α, tumor necrosis factor alpha; Arrow (→): The arrow indicates the sequential cascade of events in the pyroptotic pathway.

**Table 3:** Overview of therapies targeting inflammasomes and pyroptosis in AA.

Therapy/Agent	Main Target in the Pyroptosis Pathway	Evidence in AA (Model/LoE)	Notes
JAK inhibitors	Lower IFN-γ and IL-15 → reduced NF-κB activation and limited inflammasome formation; indirectly lower IL-1β/IL-18 production and caspase-1 activation	Human trials (LoE-H2); animal AA-specific models (C3H/HeJ mice) (LoE-A2)	Effective AA therapies; data on topical formulations are mixed
Biologics	Blocking Th2/IL-12/23/IL-17 pathways → can change the cytokine environment that activates inflammasomes	Human data heterogeneous (LoE-H2/H1)	Responses inconsistent; rare reports of AA induction with dupilumab; need controlled studies and biomarker stratification (e.g., IgE).
PDE4 inhibitors	Increase cAMP → suppress NF-κB → reduce priming of NLRP3 and pro-IL-1β	Human pilot studies/case reports (LoE-H2)	Mixed efficacy in humans
Other AA-linked modulators (Abatacept, PRP, Statins, Antihistamines, Dithranol)	↓ IFN-γ/TNF or NF-κB/JAK-STAT; ↓ ROS; potential reduction of IL-1β	Human open-label/RCT/small series (LoE-H2/H1)	Mostly indirect effects on pyroptosis
Mesenchymal stem cell therapy	Anti-inflammasome action in ORS cells (↓ NLRP3/caspase-1); reduces IL-1β/IL-18	Ex vivo human models (LoE-X); animal AA-specific models (C3H/HeJ mice) (LoE-A2)	Promising but preliminary
MCC950	Direct NLRP3 inhibitor—blocks NLRP3→caspase-1 activation → prevents GSDMD cleavage	Animal AA-specific models (C3H/HeJ mice) (LoE-A2)	No human AA trials yet
Natural anti-inflammatory compounds affecting NLRP3 axis (TGP, WGBP, XZD)	↓ caspase-1 activity and IL-1β/IL-18	Animal AA-specific models (C3H/HeJ mice) (LoE-A2); clinical observation s for XZD	Encouraging preclinical data; human validation needed
SCD-153	Itaconate derivative → suppresses inflammatory gene expression (IL-1β, IL-6, TLR3, IFNβ) → limits inflammasome signaling	In vitro keratinocyte studies and a non-AA (C57BL/6) hair growth model (LoE-C/A1)	No AA-specific animal or human trials yet.

AA, alopecia areata; cAMP, cyclic adenosine monophosphate; GSDMD, gasdermin D; IFN-γ, interferon gamma; IgE, immunoglobulin E; IL, interleukin; JAK, Janus kinase; LoE, level of evidence; LoE-A1, animal model not specific to AA; LoE-A2, animal model specific to AA (e.g., C3H/HeJ mice); LoE-C, in vitro/cell culture studies; LoE-H1, human randomized controlled trials; LoE-H2, human observational studies, case series, or pilot studies; LoE-X, ex vivo human tissue models; MCC950, selective NLRP3 inflammasome inhibitor; NF-κB, nuclear factor kappa B; NLRP3, NOD-like receptor family pyrin domain containing 3; ORS, outer root sheath; PDE4, phosphodiesterase 4; PRP, platelet-rich plasma; RCT, randomized controlled trial; ROS, reactive oxygen species; STAT, signal transducer and activator of transcription; TGP, total glucosides of paeony; Th, T helper cell; TLR3, Toll-like receptor 3; WGBP, Wugen Bushen Prescription; XZD, Xiaoji Zhiyang Decoction. Arrow (→): The arrow indicates the sequential cascade of events in the pyroptotic pathway. Arrow (↓): The arrow indicates the decrease.

## Data Availability

Not applicable.

## Abbreviations

The following abbreviations are used in this manuscript:

AA	Alopecia Areata
IL	Interleukin (np. IL-1β, IL-10, IL-12 itd.)
PCD	Programmed cell death
GSDMD	Gasdermin D
ASM	Acid sphingomyelinase
PRR	Pattern recognition receptor
PAMP	Pathogen-associated molecular pattern
DAMP	Damage-associated molecular pattern
NLRP	NOD-like receptor family pyrin domain containing protein
ASC	Apoptosis-associated speck-like protein containing a CARD
LPS	Lipopolysaccharide
AIM2	Absent in melanoma 2
ATP	Adenosine triphosphate
DNA	Deoxyribonucleic acid
NF-κB	Nuclear Factor kappa-light-chain enhancer of activated B cells
TLR	Toll-like receptor
MyD88	Myeloid differentiation primary response 88
TRIF	TIR-domain-containing adapter-inducing interferon-β
IRAK	Interleukin-1 receptor–associated kinase
IFN	Interferon
NK	Natural Killer cell
GZMA/GZMB	Granzyme A/Granzyme B
HF	Hair follicle
MHC	Major histocompatibility complex
TGF-β1	Transforming growth factor beta 1
α-MSH	Alpha-melanocyte-stimulating hormone
IDO	Indoleamine 2,3-dioxygenase
VIP	Vasoactive intestinal peptide
NKG2D	Natural Killer Group 2 Member D
Th	T helper (Th1, Th2, Th17)
TNF	Tumor necrosis factor
CXCL	Chemokine (C-X-C motif) ligand (CXCL9, CXCL10, CXCL11)
CXCR3	C-X-C chemokine receptor type 3
JAK	Janus kinase
STAT	Signal transducer and activator of transcription
ORS	Outer root sheath
C3H/HeJ	C3H substrain of inbred mice carrying the Lps^d mutation (defective Toll-like receptor 4, TLR4)
RNA	Ribonucleic acid
PTEN	Phosphatase and tensin homolog
PINK1	PTEN-induced kinase 1
ROS	Reactive oxygen species
PDE4	Phosphodiesterase 4
cAMP	Cyclic adenosine monophosphate
PRP	Platelet-rich plasma
hORSCs	Human outer root sheath cells
MSCT/MSC	Mesenchymal stem cell therapy/Mesenchymal stem cells
FGF	Fibroblast growth factor (FGF2, FGF7)
TGP	Total glucosides of paeony
WGBP	Water-soluble Ginkgo biloba leaf polysaccharides
VEGF	Vascular endothelial growth factor
HGF	Hepatocyte growth factor
XZD	Xuefu Zhuyu decoction
4-MI	4-methyl itaconate
